# Correlations Between the Inherent Components of Grains in Various Rice Varieties and the Quality of Sweet Rice Wine

**DOI:** 10.3390/foods15010179

**Published:** 2026-01-05

**Authors:** Xia Zhao, Pingyun Duan, Caixia Fan, Xinyue Wang, Jiuyuebumo Su, Xuelian Wang, Xingyu Li, Zuoling Wang, Yue Peng

**Affiliations:** 1 Faculty of Agriculture, Forestry and Food Engineering, Yibin University, Yibin 644000, China; 2 Solid-State Fermentation Resource Utilization Key Laboratory of Sichuan Province, Yibin University, Yibin 644000, China; 3 Key Laboratory of Aromatic Plant Resource Exploitation and Utilization in Sichuan Higher Education, Yibin University, Yibin 644000, China

**Keywords:** rice varieties, sweet rice wine, starch, rice components

## Abstract

The inherent chemical composition of different rice varieties can significantly influence the quality of sweet rice wine. However, most studies on sweet rice wine overlook varietal characteristics, resulting in slow progress in breeding rice varieties specialized for sweet rice wine production. To investigate the relationship between the inherent chemical composition of various rice varieties, such as starch, protein, and crude fat content, and their corresponding rice wines, 16 rice varieties with significant compositional variation were used in this study. The results revealed that screening solely for glutinous or non-glutinous rice is insufficient to select suitable raw materials for sweet rice wine production. Correlation analysis showed that the total sugar content of sweet rice wine was primarily associated with starch properties. In contrast, the formation of alcoholic strength and juice yield was more complex, exhibiting close correlations with multiple rice components, including amylose, albumin, globulin, crude fat, tannin content, and others. Furthermore, interactions among these components were also significantly correlated with these quality traits. In conclusion, amylose content, the ratio of amylose to amylopectin, gel consistency, and albumin content are important indicators for the rapid screening of high-quality rice lines, as they strongly correlate with sweet rice wine quality. These results will facilitate the development of rice varieties specialized for sweet rice wine production.

## 1. Introduction

Sweet rice wine is a popular alcoholic beverage in China and has been an essential part of the cultural and spiritual life of the Chinese people for thousands of years. It is also known for its sweet flavor, delicate aroma, mild taste, and rich nutritional composition [1]. It is generally produced through semisolid-state fermentation of rice. During this process, rice kernels are cooked, saccharified, and fermented. Throughout fermentation, macromolecular substances in the rice are broken down and catabolized by microorganisms, such as proteins, crude fat, and starch. This fermentation process not only increases the sensory qualities but also increases the nutritional value of sweet rice wine, meeting the requirements for functional foods with high health benefits [2,3,4].

It is well known that the complex flavor components in rice wine are closely related to the metabolism of various microorganisms and the fermentation conditions during traditional brewing. Thus, in recent years, large numbers of studies have focused on the microbial community and environmental factors [5,6,7]. However, rice primarily provides substrates for microbial metabolism and influences the components and flavor of sweet rice wine [2,8]. Rice is rich in starch (70–80%), which comprises two types of molecules: amylose and amylopectin. Amylose is an unbranched helical molecule, whereas amylopectin is a highly branched glucose polymer. Rice is classified as either non-glutinous or glutinous based on its amylose content [9,10]. Glutinous rice varieties contain very little amylose (typically about 0 to 4%), whereas non-glutinous rice varieties naturally contain as much as 30% amylose [10]. Due to differences in starch composition between non-glutinous and glutinous rice, distinct starch structures have been observed in sweet rice wines and their vinasses derived from the two rice types [1]. Lai et al. [11] suggested that sweet rice wine fermented from glutinous rice is superior to that from non-glutinous rice in taste and texture. Rice also contains different protein constituents, including glutelin, albumin, globulin, and gliadin [12]. Chen et al. [13] reported that exogenous glutelin addition promoted total alcohol production and was significantly associated with umami, honey, and fruity flavors; exogenous albumin was closely related to bitter and astringent flavors. Fat is abundant in the epidermal layer of the rice kernel [14]. Few studies have focused on the effect of fat on rice wine fermentation. However, previous research demonstrated that modulating unsaturated fatty acid content was a potential way to increase ester production and enhance the aroma of red wine [15].

Although a lot of researchers have studied the effects of various chemical constituents on the flavor characteristics of sweet rice wine, most of them have only focused on the addition of exogenous starch, protein, or hydrolase. Furthermore, experimental materials often lacked clear varietal information or were directly purchased from supermarkets [4,11,13,16]. The impact of varietal discrepances on the fermentation process and wine quality has been observed in kiwifruit wine [17] and grape wine [18]. Wang et al. [19] also reported differences among rice varieties in the formation of higher alcohols during rice wine fermentation, based on ten rice varieties from two production regions. Therefore, we hypothesize that the inherent chemical composition profiles of different varieties significantly influence the quality of sweet rice wine, and that strong correlations exist between these components and the wine’s quality. However, these relationships have not yet been systematically investigated.

In recent years, rice quality, especially its eating quality, has been a major focus. Enhancing the eating quality of rice while maintaining high yield is crucial for rice breeding in China. Along with the extensive development of rice cultivar, high yield and the eating quality rice cultivars have emerged in large numbers, while varieties owing significant discrepance on compositions were also presented [20,21,22]. Different varieties exhibit distinct quality properties primarily due to variations in the percentage and structure of macromolecular components, including proteins, fatty acid, and starch [9,23]. Additionally, the interactions among these components can significantly influence the eating quality of rice [24]. However, grain quality is a complex trait because it is determined by the interaction among many grain components. High eating quality does not necessarily correspond to high quality for sweet rice wine fermentation or other processing industries. Thus, many breeding departments have attempted to breed a series of special-purpose rice varieties to meet the diverse demands for rice quality traits in different areas [25]. However, the quality characteristics of rice varieties suitable for fermenting sweet rice wine remain unclear, and reliable indicators for rapid screening of breeding materials are lacking. As a result, the breeding progress of special-purpose rice varieties has been slow. To address this issue, 16 rice varieties with different quality characteristics from southern China were used in this experiment to uncover the inherent compositional profiles of different varieties, such as starch, protein, and crude fat content, and the correlations between these rice kernel components and the main quality characteristics of sweet rice wine. Moreover, evaluation indicators for varietal screening were raised. These results will facilitate the rapid selection of breeding materials and the development of rice varieties specialized for sweet rice wine production, further improving the quality of sweet rice wine.

## 2. Materials and Methods

### 2.1. Experiment Materials

**Figure A1:**
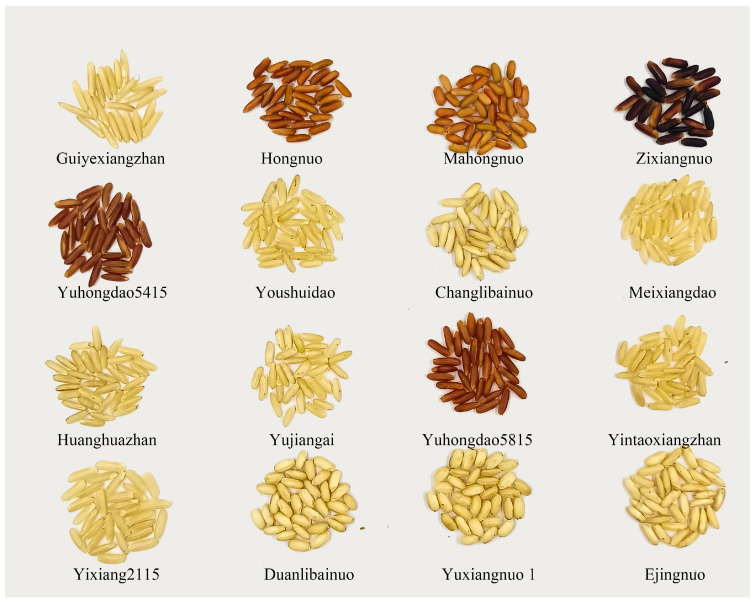
Grain morphology of different rice varieties used in this experiment.

This research was conducted using sixteen rice varieties, the details of which are presented in Table A1 and grain morphology of each variety is shown in Figure A1. A field experiment was conducted in 2023 in Fuxing Village, Fushun County. The experiment was designed as a randomized complete block design with three biological replications [26]. The plot area of each biological replicate was 20 m^2^. The experimental site is located at southern China and has a subtropical humid monsoon climate. Seedlings were transplanted on 10 April and harvested at maturity. After harvest, rice grains were air-dried in the natural environment, and the moisture content of brown rice was adjusted to 14% for subsequent experiments.

### 2.2. Components of the Rice Kernel

The kernels from each biological replication were hulled using an electric dehusker (JLGJ-4.5, Taizhou Cereal and Oil Instrument Co. Ltd., Taizhou, China) to obtain brown rice. Some rice samples (15 g) were then ground for 20 s using a grinder (JXFSTPRP-24L Shanghai Jinsin Industrial Development Co., Ltd., Shanghai, China), and the resulting flour was filtered through a 100-mesh standard sieve before being used for analysis, while some rice samples (100 g) were used for sweet rice wine fermentation.

#### 2.2.1. Determination of Amylose, Amylopectin, and Total Starch Content

The content of amylopectin, amylose, and total starch (%, dry basis) in rice grains were measured by the method of Govindaraju et al. [27], with some modifications. One hundred milligrams of rice flour were dispersed in 9 mL of 1 mol L^−1^ KOH. The mixture was then incubated at 85 °C for 20 min, and then cooling to ambient temperature. The sample solution (1 mL) was pipetted into a 50 mL beaker. The solution was titrated with 1% HCl to pH 3. Subsequently, 0.5 mL of 0.2% iodine solution (2.0 g of potassium iodide and 0.2 g of iodine were dissolved in 100 mL of distilled water) was added. deionized water was used to adjusted the volume of the mixture to 50 mL, and then it was placed in darkness for 20 min. A control solution was prepared in the same manner but without the sample. Absorbances measured at 449 and 596 nm were used to calculate amylose content by reference to an amylose standard curve, while absorbances at 533 and 705 nm were used to calculate amylopectin content by reference to an amylopectin standard curve.

Total starch content = amylose content + amylopectin content.

#### 2.2.2. Determination of Gel Consistency

The gel consistency of each sample was determined using two technical replicates, following the method described by Zeng et al. [21]. A 100 mg flour sample was placed in a tube and 0.2 mL of 0.025% thymol blue was added to dispersed and dissolved it. Subsequently, 2.0 mL of 0.2 mol L^−1^ KOH was pipetted into the tubes. Each tube was then covered with a glass marble and kept in boiling water for 8 min. After boiling water bath, all tubes were cooled down in ice water for 20 min, and then laid horizontally in an incubator set at 20 °C. The length of the rice gel in the tube was measured once the gel ceased flowing.

#### 2.2.3. Determination of Protein, Crude Fat and Tannin Content

Soluble proteins were classified as glutelin, albumin, globulin, and prolamin. They were successively extracted using 9 mL of distilled water, 5% sodium chloride, 70% ethanol, and 0.2% sodium hydroxide, following the method of Yang et al. [4]. A 200 mg flour sample was placed in a tube, and 9 mL of the above extract solution was added to disperse it. The mixture was shaken at 30 °C for 2 h. The protein content in the extracts was determined at room temperature using the Coomassie Brilliant Blue G-250 assay (Hefei Bomei Biotechnology Co., Ltd., Hefei, China) (100 mg of Coomassie Brilliant Blue G-250 powder was dissolved in 50 mL of 90% ethanol. Then, 100 mL of 85% phosphoric acid was added. The mixture was diluted to a final volume of 1000 mL with distilled water). The absorbance of the reaction mixture was measured at 595 nm within 1 h. Two technical replicates were used to determine the protein concentration. Crude fat content was measured with a crude fat analyzer (SZF-06C, Zhejiang Top Cloud Agriculture Co., Ltd., Hangzhou, China). The content of tannin was measured in accordance with the Chinese national standard [28].

### 2.3. Sweet Rice Wine Fermentation in Lab-Scale

The fermentation of sweet rice wine was generally performed in five main steps according to the method of Cai et al. [5]. First, one hundred grams of rice from a single biological replicate were soaked in 250 mL of distilled water for 24 h at 25 °C, followed by rinsing twice and steaming for 28 min to gelatinize the starch. The cooked rice was then cooled to 30 °C in a 500 mL bottle and then added 0.17 g of starter (Angel rice leaven) to each sample. The samples were incubated at 30 °C for 24 h, then at 25 °C for 72 h. After fermentation mixture was centrifuged at 5000 rpm for 5 min at ambient temperature, rice wine liquid of each sample was collected. The volume of the supernatant was defined as the juice yield. Supernatants and solid insoluble precipitates were stored at 4 °C until analysis.

### 2.4. Physicochemical Properties of Sweet Rice Wine

Above rice wine liquids of each variety, with three biological replicates, were analyzed for pH, alcohol content, and total sugar content. The pH levels were determined using a pH Benchtop meter (PHSJ-3F, Shanghai Yifen Scientific Instruments Co., Ltd., Shanghai, China). Total sugars were determined by the anthrone method, with glucose used as the standard. Alcohol content was measured using the potassium dichromate colorimetric method.

### 2.5. Statistical Analysis

The differences among groups were analyzed using two-way analysis of variance (ANOVA), followed by Duncan’s multiple range test (*p* < 0.05). Principal component analysis (PCA) of rice components and the quality of sweet rice wine was conducted using Origin 2024. Correlation analysis was performed using the Pearson method in Origin 2024.

## 3. Results

### 3.1. Components and Property of Different Rice Varieties

**Figure 1:**
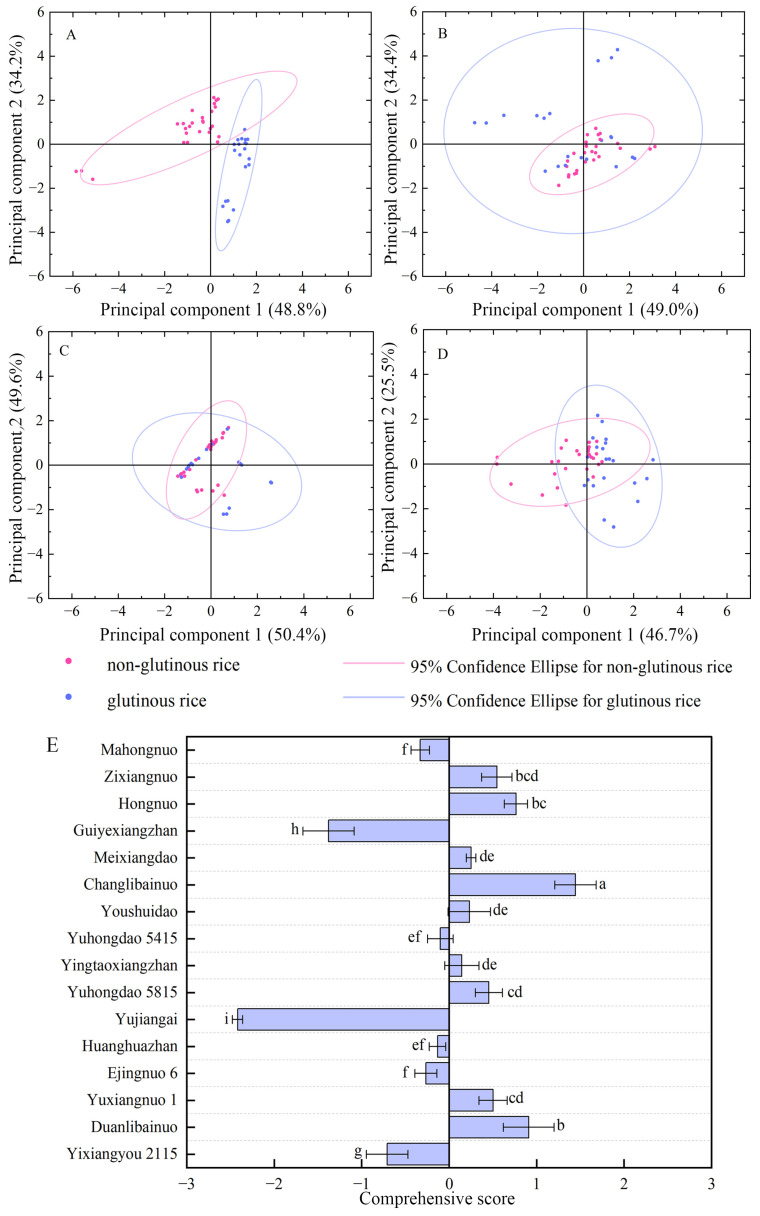
Score plots of principal component analysis (PCA). (**A**) PCA of starch components and properties in the grains of sixteen rice varieties; (**B**) PCA of protein components in the grains of sixteen rice varieties; (**C**) PCA of tannin and crude fat content in the grains of sixteen rice varieties; (**D**) PCA of the quality of sweet rice wine fermented from brown rice of sixteen rice varieties; (**E**) Comprehensive scores of sixteen rice varieties. Lowercase letters indicate significance based on ANOVA results (*p* < 0.05).

Different rice varieties exhibited significant discrepancies in grain morphology (Figure A1). The amylose and amylopectin contents, as well as their respective proportions, also showed notable variation (Table 1). The amylose content in non-glutinous rice varieties varied by up to 14.87%, whereas in glutinous rice varieties, it was less than 3.23%. Among the glutinous varieties, Ejingnuo 6 had the highest amylose content. The variation in amylopectin content reached 46.18%. The non-glutinous variety Yujiangai had the highest amylose content and the lowest amylopectin content. Apart from Yujiangai, the glutinous varieties Zixiangnuo and Mahongnuo also exhibited lower amylose, amylopectin, and total starch contents compared to other varieties. These results contribute to the significant differences observed in the percentages of amylose and amylopectin. Starch composition plays a key role in gel consistency, which varied by up to 12.1 cm. Glutinous varieties generally exhibited relatively high gel consistency; however, the difference in gel consistency between some non-glutinous varieties, such as Guiyexiangzhan and Meixiangdao, and glutinous varieties was less pronounced. Although non-glutinous and glutinous rice varieties exhibited significant differences in amylose content, PCA revealed only slight differences in starch characteristics, as evidenced by the overlap observed in the score plot (Figure 1A).

A significant difference in protein content was observed among these varieties. Glutelin is the primary protein in rice, with its content varying by as much as 10.65 mg/g. Hongnuo had the lowest glutelin and total protein content, whereas the glutinous varieties Zixiangnuo and Yujiangai exhibited the highest levels of glutelin and total protein. Although albumin, globulin, and gliadin were present at relatively low concentrations, their levels varied noticeably among the varieties, with differences of 1.98 mg/g, 4.33 mg/g, and 1.84 mg/g, respectively. PCA of protein content showed that glutinous rice varieties exhibited greater variance in protein content compared to non-glutinous varieties; however, only a slight difference in PCA scores was observed between the two groups (Figure 1B).

Colored rice had significantly higher tannin content compared to white rice. A significant difference was also observed among colored rice varieties, whereas no significant difference was found among white rice varieties. Crude fat content varied by as much as 2.06%. Yujiangai and Yuhongdao 5815 had the lowest crude fat content, while Yintaoxiangzhan and Zixiangnuo exhibited the highest. The percentages of total starch, total protein, and crude fat differed significantly between varieties. PCA of tannin and crude fat content revealed only slight differences in PCA scores between non-glutinous and glutinous rice varieties (Figure 1C).

### 3.2. Primary Quality of Sweet Rice Wine

Sweet rice wine was produced through the fermentation of various rice varieties. Table 2 shows significant differences in total sugar content, alcoholic strength, and juice yield among these varieties. The variations in total sugar, alcoholic strength, and juice yield were 13.22 g 100 mL^−1^, 0.19, 7.77%, and 32.47 mL 100 g^−1^, respectively. No notable difference in juice pH was observed. Two principal components were identified through the PCA of these quality components, with contribution rates of 46.67% for principal component 1 and 25.45% for principal component 2 (Figure 1D). To assess the suitability of each variety for sweet rice wine production, a comprehensive quality score was calculated using PCA (Figure 1E). Five glutinous rice varieties achieved the highest scores, including Changlibainuo, Duanlibainuo, Zixiangnuo, Hongnuo, and Yuxiangnuo 1. In contrast, two glutinous rice varieties, Mahongnuo and Ejingnuo 6, had relatively lower scores. No significant difference in comprehensive scores was found between some non-glutinous rice varieties and certain high-scoring glutinous rice varieties, such as Yuhongdao 5815, Meixiangdao, Youshuidao, and Yintaoxiangzhan. The overlap in scores between non-glutinous and glutinous rice varieties observed in the score plot indicated minimal differences in rice wine quality between these two rice types.

### 3.3. Correlation Between Rice Components and Main Quality Components of Sweet Rice Wine

**Figure 2:**
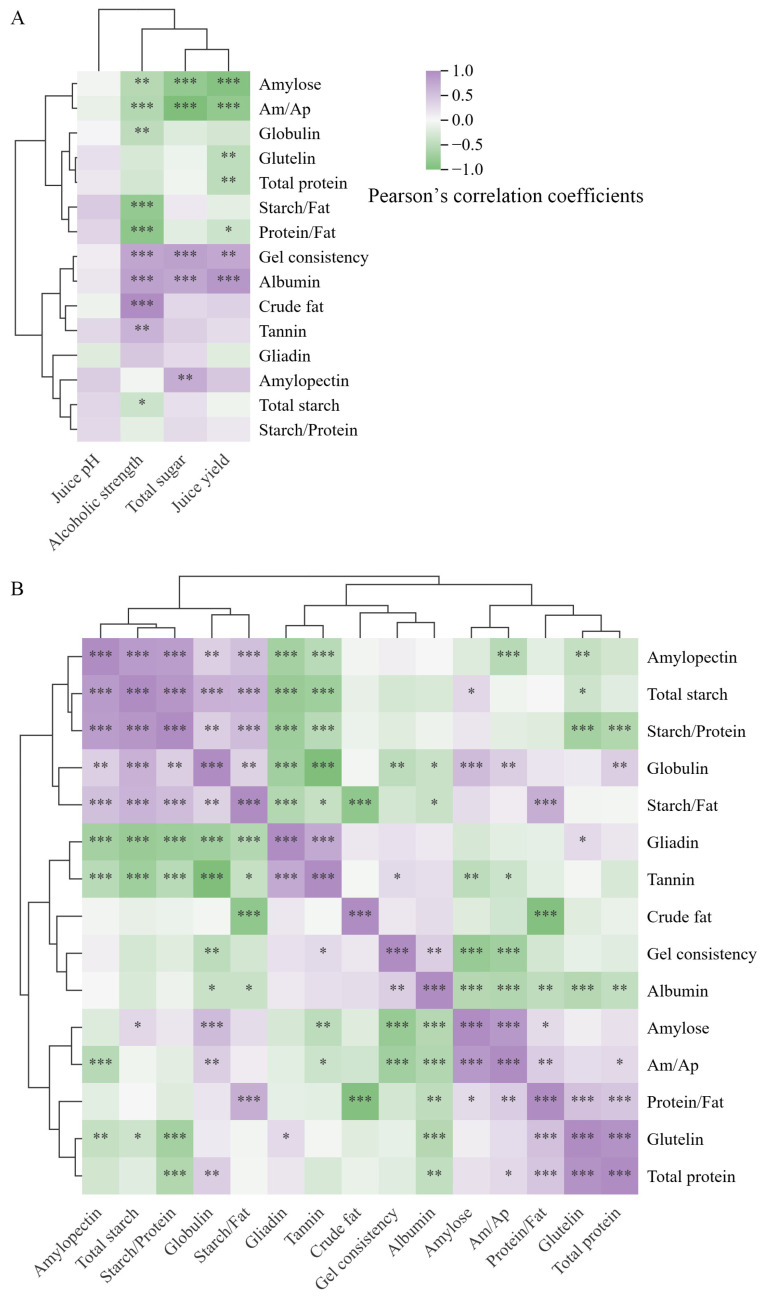
Cluster correlation analysis heatmap between the components of sweet rice wine and rice (**A**) and cluster correlation analysis heatmap among the components of rice (**B**). *, **, and *** denote significant differences at the 0.05, 0.01, and 0.001 levels of significance, respectively. The colors represent Pearson’s correlation coefficients. Am/Ap denotes the ratio of amylose to amylopectin content.

The results of the correlation analysis are shown in Figure 2. The total sugar content of sweet rice wine was positively correlated with amylopectin content, gel consistency, and albumin content, while it was negatively correlated with amylose content. Alcoholic strength was significantly influenced by various rice components and properties. It showed positive correlations with gel consistency, albumin, crude fat, and tannin content, and negative correlations with amylose, total starch, globulin, as well as with ratios among components such as amylose/amylopectin (Am/Ap), protein/fat, and starch/fat. Juice yield exhibited significant positive correlations only with gel consistency and albumin content, while showing significant negative correlations with amylose, glutelin, total protein content, Am/Ap, and protein/fat. No significant correlation was found between rice components and juice pH. These components of rice also showed closely correlations with each other (Figure 2B).

### 3.4. Morphology and Diameter of Starch Granule

**Figure 3:**
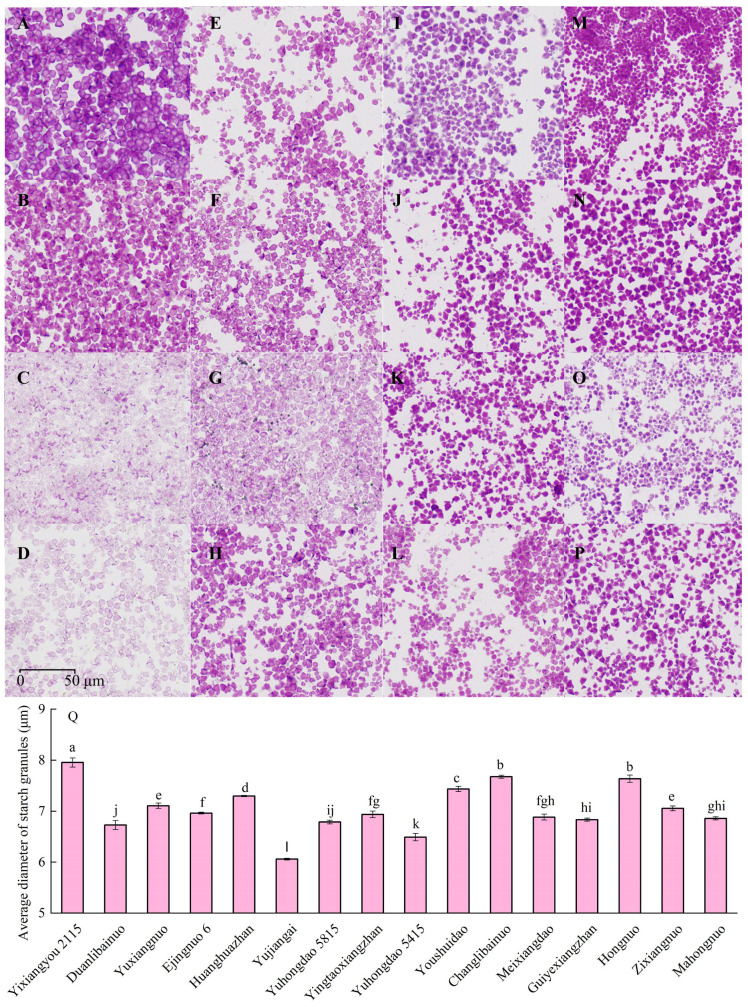
Morphology and diameter of starch granules in the grains of various rice varieties. (**A**): Yixiangyou 2115; (**B**): Huanghuazhan; (**C**): Yujiangai; (**D**): Yuhongdao 5815; (**E**): Yintaoxiangzhan; (**F**): Yuhongdao 5415; (**G**): Youshuidao; (**H**): Guiyexiangzhan; (**I**): Hongnuo; (**J**): Zixiangnuo; (**K**): Mahongnuo; (**L**): Meixiangdao; (**M**): Duanlibainuo; (**N**): Yuxiangnuo; (**O**): Ejingnuo; (**P**): Changlibainuo. (**Q**): The average diameter of starch granules of various rice varieties. Lowercase letters indicate significance based on ANOVA results (*p* < 0.05).

**Figure 4:**
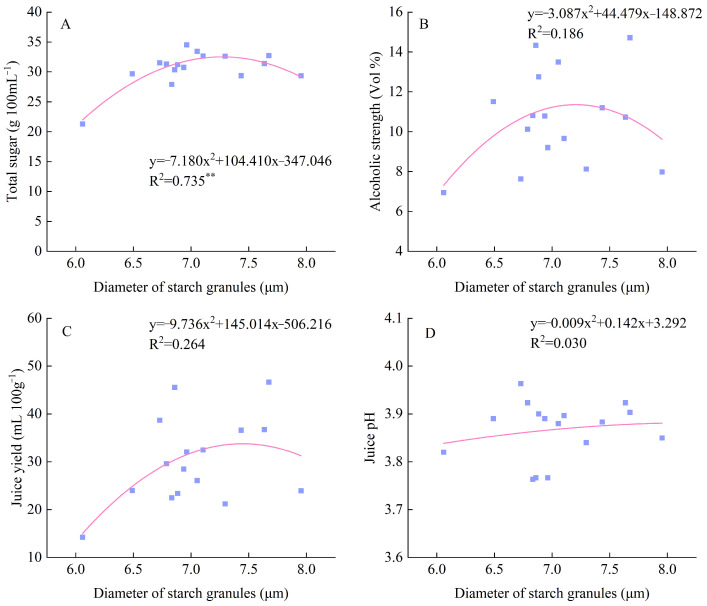
Quadratic regression relationship between starch granule diameter and total sugar (**A**), alcoholic strength (**B**), Juice yield (**C**), and Juice pH (**D**) of sweet rice wine. “**” denotes a significant difference at the 0.01 level of significance.

Figure 3 shows the starch granule morphology of different rice varieties. Most glutinous rice varieties exhibited angular starch granules, such as Changlibainuo, Yuxiangnuo 1, Hongnuo, and Zixiangnuo. In contrast, most non-glutinous rice varieties had relatively round starch granules, including Yixiangyou 2115, Huanghuazhan, and Yuhongdao 5415. A significant difference in starch granule diameter was observed. Yixiangyou 2115 had the largest starch granule diameter, while Yujiangai had the smallest. Additionally, there was a significant quadratic regression relationship between starch granule diameter and the total sugar content of sweet rice wine, with a regression coefficient of 0.74. However, no significant regression relationship was found between starch granule diameter and juice pH, alcoholic strength, or juice yield (Figure 4).

## 4. Discussion

### 4.1. Different Rice Varieties Exhibit Significant Variations in Quality Components

China is the largest single national consumer and producer of rice and is also the birthplace of rice cultivation. A rich diversity of rice varieties has been found in China. In recent years, along with the development of rice varieties, numerous high yield and high eating quality rice varieties have emerged [20,21,22]. The constituents of the rice endosperm contained starch, fats, proteins, amino acids, vitamins, and other secondary metabolites. Among these, starch, fats and proteins contribute approximately 80% of the dry matter weight of the rice kernel [20]. Therefore, nine non-glutinous and seven glutinous rice varieties, each possessing distinct quality characteristics, were analyzed to reveal variations in starch, protein, crude fat, and tannin content (Table 1). Different varieties exhibited significant discrepances in amylose and amylopectin content and their respective proportions. The variation in amylose content among non-glutinous rice varieties reached 14.87%, while the amylose content in glutinous rice varieties was less than 3.23%. The variation in amylopectin content reached 46.18%. These results contributed to the significant differences observed in the percentages of amylose and amylopectin. The starch composition plays a key role in gel consistency and starch granule structure [10]. The variation in gel consistency and starch granule diameter reached 12.1 cm and 1.90 μm, respectively. The Waxy/GBSSI genes, which encode granule-bound starch synthase, control amylose synthesis and account for apparent amylose content within a range of 0 to 30% [29]. The significant variations in the content and composition of starch were consistent with the findings of Farooq et al. [30]. A significant difference in protein content was also observed. Glutelin is the primary protein in rice and structural properties of it seriously impacts the formation of grain quality [31]. Its content varied by as much as 10.65 mg/g. Albumin, globulin, and gliadin contents varied significantly among the varieties, despite their overall low concentrations. These results are consistent with the study of Li et al. [12]. Colored rice had significantly higher tannin content compared to white rice. Significant differences in tannin content were also observed among colored rice varieties, whereas no significant differences were found among white rice varieties. Crude fat content also exhibited significant variation, reaching as much as 2.06%. Although rice kernels contain relatively low levels of crude fat and protein, correlation analysis (Figure 3B) revealed a strong relationship between these components and starch, indicating interactions among them. These interactions play a crucial role in the structure of starch granules and the overall quality of rice [14,15]. The significant variance in diameter of starch granules also observed in this study. Although non-glutinous and glutinous rice varieties exhibited significant differences in amylose content, PCA revealed only slight differences in starch characteristics, tannin and crude fat content. Furthermore, PCA of protein content showed that glutinous rice varieties exhibited higher variance in protein composition compared to non-glutinous varieties; however, only minor difference in PCA scores was also found between them.

### 4.2. Main Quality Components of Sweet Rice Wine Are Closely Correlated with Rice Components

Although a lot of researchers have studied the effects of various chemical constituents on the flavor characteristics of sweet rice wine, most of them have only focused on the addition of exogenous starch, protein, or hydrolase. Furthermore, experimental materials often lacked clear varietal information or were directly purchased from supermarkets [4,11,13,16]. The inherent chemical composition of various rice varieties and their influence on sweet rice wine were overlook. Several studies have detailed the flavor components and properties of different rice wines [32,33,34], while Inoue et al. [35] and Wang et al. [36] revealed discrepancies in the quality of rice wine produced from different rice varieties as raw materials. This study also obtained similar results. Furthermore, to evaluate the suitability of each variety for sweet rice wine production, a comprehensive score of sweet rice wine quality was calculated via PCA. Five glutinous rice varieties achieved the highest scores, including Changlibainuo, Duanlibainuo, Zixiangnuo, Hongnuo, and Yuxiangnuo 1. Chinese sweet rice wine has traditionally been produced using only glutinous rice [4]. Glutinous rice is a superior commercial raw material for sweet rice wine fermentation compared to non-glutinous rice, for superior in taste and texture [11]. However, two glutinous rice varieties, Mahongnuo and Ejingnuo 6, received relatively lower scores. No significant variance in comprehensive scores was found between some non-glutinous rice varieties and certain high-scoring glutinous rice varieties, such as Yuhongdao 5815, Meixiangdao, Youshuidao, and Yintaoxiangzhan (Figure 1). The overlap in scores between non-glutinous and glutinous rice varieties observed in the score plot indicated no significant difference between these two types. This finding also highlights the important role of other rice components in rice wine fermentation beyond amylose content, including protein constituents, and crude fat, which also reported by other studies [37]. PCA also revealed only slight differences in starch characteristics, tannin and crude fat content, protein composition of rice between these two types. Therefore, screening solely for non-glutinous or glutinous rice is insufficient to identify suitable raw materials for sweet rice wine production. The interactions among these components in the grains identified in this study may contribute to this phenomenon. Consideration of the different constituent contents and ratios in rice varieties is essential. At the same time, the relationship between the inherent composition of these varieties and the primary quality characteristics of sweet rice wine needs to be fully investigated. The total sugar content of sweet rice wine showed a positive correlation with amylopectin content, gel consistency, and albumin content, while it was negatively correlated with amylose content. Additionally, it exhibited a significant quadratic regression relationship with the diameter of starch granules. Alcoholic strength was positively correlated with gel consistency, albumin, crude fat, and tannin content; conversely, it was negatively correlated with amylose, total starch, and globulin, as well as with ratios among components such as Am/Ap, protein/fat, and starch/fat. Juice yield had significant positive correlations only with gel consistency and albumin content, while showing significant negative correlations with amylose, glutelin, total protein content, Am/Ap, and protein/fat. Wang et al. [2] demonstrated that a significant positive correlation between the main differential volatile aroma compounds in Chinese rice wine and the starch and fat content of glutinous rice across four varieties. Wang et al. [24] reported that starch in rice was strongly correlated with higher alcohols in rice wine based on a study of 10 rice varieties. High eating quality does not necessarily correspond to high quality for fermentation in the production of sweet rice wine or other processing industries [38]. Reliable indicators for the rapid screening of breeding materials are essential for developing rice varieties specialized for sweet rice wine production. Based on these correlations, amylose content and Am/Ap were identified as negative indicators, while gel consistency and albumin content were recognized as notable positive indicators for the rapid screening of high-quality rice lines. Because all of them showed strong correlations with total sugar content, alcoholic strength, and juice yield of sweet rice wine. But Chen et al. [13] reported that exogenous albumin was closely related to bitter and astringent flavors. The source and concentration of albumin may contribute to this discrepancy. Besides these indicators, the interaction of components in rice was identified in this study and should not be overlooked in practical applications. The effect of this interaction on sweet rice wine warrants further investigation in the future. Not only do the results support the breeding of rice varieties specialized for fermenting sweet rice wine, but the research methods also provide valuable guidance for developing special-purpose other crop varieties that meet diverse quality trait requirements across various food production.

## 5. Conclusions

**Figure 5:**
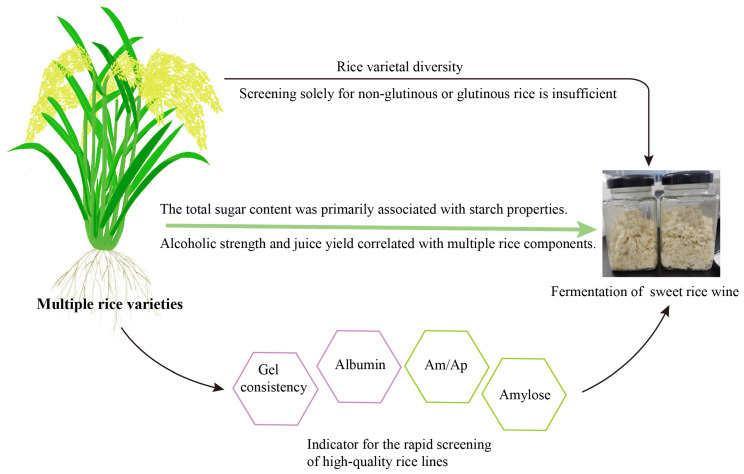
Schematic diagram illustrating the regulation of rice variety characteristics on the quality of sweet rice wine. Note: Black arrows indicate influence. Green arrow indicates correlation. Pink hexagons represent positive indicators, while green hexagons represent negative indicators. Am/Ap denotes the ratio of amylose to amylopectin content.

Different rice varieties exhibit significant discrepancies in grain morphology, starch, protein, crude fat, and tannin content. Correlation analysis in this study indicated that the total sugar content of sweet rice wine was primarily associated with starch properties. There was a significant quadratic regression relationship between starch granule diameter and the total sugar content of sweet rice wine. In contrast, the formation of alcoholic strength and juice yield was more complex, showing close correlations with multiple rice components. Furthermore, the proportions of these components were also significantly correlated with the alcoholic strength and juice yield of sweet rice wine. These results establish a link between grain components of various genotype and quality of sweet rice wine. Breeding specialized rice varieties is essential for producing sweet rice wine. Based on these correlations, amylose content and Am/Ap were identified as negative indicators, while gel consistency and albumin content were recognized as notable positive indicators for the rapid screening of high-quality rice lines. Because all of them showed strong correlations with total sugar content, alcoholic strength, and juice yield of sweet rice wine. (Figure 5). These findings will lay the foundation for breeding rice varieties specialized for the fermentation of sweet rice wine and for establishing an evaluation system for its raw materials, thereby further improving the quality of sweet rice wine.

## Figures and Tables

**Table 1:** Components and their ratio in the grains of different rice varieties.

Variety	Gel Consistency cm	Amylose %	Amylopectin %	Total Starch %	Am/Ap	Glutelin mg g^−1^	Albumin mg g^−1^	Globulin mg g^−1^	Gliadin mg g^−1^	Total Protein mg g^−1^	Tannin mg g^−1^	Crude Fat %	Starch/ Protein	Starch/Fat	Protein /Fat
Yixiangyou 2115	3.83 g	12.20 e	79.01 ab	91.30 ab	0.154 e	25.58 efg	2.94 e	5.12 d	1.277 ghi	34.92 bc	0.17 fg	3.98 c	26.18 ab	23.24 def	0.89 f
Duanlibainuo	11.8 cd	2.45 f	79.30 ab	81.76 def	0.031 f	30.71 ab	2.81 e	5.37 c	0.96 k	29.84 a	0.15 fg	2.79 ef	20.55 gh	29.34 b	1.43 a
Yuxiangnuo 1	13.43 ab	2.56 f	76.05 b	78.61 f	0.034 f	29.29 bc	3.34 cd	5.14 d	1.52 de	39.29 a	0.14 fg	3.22 d	20.00 h	24.58 de	1.23 cd
Ejingnuo 6	12.80 abc	3.23 f	81.89 a	85.12 cd	0.040 f	26.10 defg	2.93 e	4.68 e	1.26 hi	34.98 bc	0.12 fg	3.11 def	24.33 bcd	25.10 bc	1.13 d
Huanghuazhan	4.5 g	17.15 b	68.07 d	85.22 cd	0.254 b	28.06 cd	3.65 bc	5.69 ab	1.77 c	39.16 a	0.21 f	2.83 def	21.77 fgh	30.09 ab	1.39 ab
Yujiangai	2.10 h	26.44 a	33.12 g	59.55 h	0.799 a	30.93 ab	2.12 f	5.44 c	1.42 ef	39.91 a	0.13 fg	2.71 f	14.92 i	20.05 ef	1.48 a
Yuhongdao 5815	7.70 e	12.04 e	76.15 b	88.19 abc	0.168 e	27.27 cdef	2.32 f	4.19 f	1.31 fgh	35.10 bc	0.73 e	2.71 f	25.20 bc	32.51 a	1.29 bc
Yingtaoxiangzhan	5.26 fg	14.15 d	70.36 cd	84.51 cde	0.201 cd	26.28 defg	3.08 de	5.47 c	1.38 fg	36.22 bc	0.11 fg	4.77 a	23.36 cdef	17.71 g	0.76 f
Yuhongdao 5415	4.83 fg	11.29 e	68.47 d	79.76 ef	0.165 de	27.91 cde	2.89 e	4.03 f	1.55 d	36.38 b	1.07 c	3.18 de	21.95 efg	25.09 cd	1.15 d
Youshuidao	6.13 f	11.71 e	81.38 a	93.09 a	0.144 e	24.09 g	3.04 de	5.55 bc	1.07 j	33.76 c	0.12 fg	4.21 bc	27.58 a	22.10 ef	0.80 f
Changlibainuo	10.9 d	2.98 f	74.68 bc	77.65 f	0.040 f	25.05 fg	3.79 ab	4.70 e	0.94 k	34.48 bc	0.12 fg	4.31 bc	22.53 def	18.19 g	0.81 f
Meixiangdao	13.23 abc	15.62 c	75.85 b	91.47 ab	0.21 c	25.67 efg	3.43 c	5.83 a	1.19 i	36.12 bc	0.14 fg	4.31 bc	25.36 bc	21.25 f	0.84 f
Guiyexiang zhan	13.30 abc	11.57 e	75.69 b	87.27 bc	0.153 e	26.47 def	2.74 e	4.97 d	1.58 d	35.76 bc	0.12 fg	4.05 c	24.30 bcd	21.54 f	0.88 f
Hongnuo	13.7 ab	2.22 f	65.78 d	67.99 g	0.034 f	21.33 h	4.10 a	1.50 i	1.71 c	28.63 d	1.61 b	2.84 def	23.81 cde	24.00 def	1.01 e
Zixiangnuo	14.20 a	0.13 g	43.01 f	43.08 k	0.003 f	31.98 a	3.03 de	2.03 h	2.78 a	39.83 a	1.84 a	4.60 ab	10.82 j	9.4 h	0.87 f
Mahongnuo	12.3 bcd	0.48 g	49.92 e	50.41 j	0.010 f	25.31 fg	4.09 a	3.26 g	1.89 b	34.55 bc	0.97 d	4.34 bc	14.59 i	11.61 h	0.80 f

Lowercase letters in the table indicate significance based on ANOVA results (*p* < 0.05). Means sharing the same letter within a column are not significantly different. Am/Ap denotes the ratio of amylose to amylopectin content.

**Table 2:** Primary components and the pH of sweet rice wine.

Variety	Total Sugar	Juice pH	Alcoholic Strength	Juice Yield
	g 100 mL^−1^		Vol%	mL 100 g^−1^
Yixiangyou 2115	29.36 e	3.85 cde	7.98 gh	23.93 fg
Duanlibainuo	31.52 bcd	3.96 a	7.63 h	38.67 b
Yuxiangnuo 1	32.67 abc	3.90 bcd	9.66 ef	32.47 cd
Ejingnuo 6	34.51 a	3.77 f	9.2 fg	32.07 cd
Huanghuazhan	32.63 abc	3.84 de	8.13 gh	21.2 g
Yujiangai	21.29 f	3.82 e	6.95 h	14.2 h
Yuhongdao 5815	29.67 de	3.89 bcd	11.51 cd	24 fg
Yingtaoxiangzhan	30.76 bcde	3.89 bcd	10.78 de	28.47 def
Yuhongdao 5415	31.3 bcd	3.92 ab	10.13 def	29.6 de
Youshuidao	29.37 de	3.88 bcd	11.2 d	36.6 bc
Changlibainuo	32.71 abc	3.91 bc	14.72 a	46.67 a
Meixiangdao	31.22 bcd	3.91 bc	12.75 bc	23.4 g
Guiyexiangzhan	27.91 e	3.76 f	10.81 de	22.47 g
Hongnuo	31.39 bcd	3.92 ab	10.73 de	36.73 bc
Zixiangnuo	30.35 cde	3.77 f	14.34 a	45.53 a
Mahongnuo	33.43 ab	3.88 bcd	13.5 ab	26.07 efg

Lowercase letters in the table indicate significance based on ANOVA results (*p* < 0.05). Means sharing the same letter within a column are not significantly different.

## Data Availability

The original contributions presented in the study are included in the. article, further inquiries can be directed to the corresponding author.

## Abbreviations

The following abbreviations are used in this manuscript:

Am/Ap	Amylose/Amylopectin
PCA	Principal component analysis

## Appendix A

**Table A1:** The information of varieties.

Number	Variety	Type	Number	Variety	Type
1	Yixiangyou 2115	Non-Glutinous rice	9	Meixiangdao	Non-Glutinous rice
2	Huanghuazhan	Non-Glutinous rice	10	Duanlibainuo	Glutinous rice
3	Yujiangai	Non-Glutinous rice	11	Yuxiangnuo 1	Glutinous rice
4	Yuhongdao 5415	Non-Glutinous rice	12	Ejingnuo 6	Glutinous rice
5	Yingtaoxiangzhan	Non-Glutinous rice	13	Changlibainuo	Glutinous rice
6	Yuhongdao 5815	Non-Glutinous rice	14	Hongnuo	Glutinous rice
7	Youshuidao	Non-Glutinous rice	15	Zixiangnuo	Glutinous rice
8	Guiyexiangzhan	Non-Glutinous rice	16	Mahongnuo	Glutinous rice
